# COVID-19 and Diabetes: A Comprehensive Review of Angiotensin Converting Enzyme 2, Mutual Effects and Pharmacotherapy

**DOI:** 10.3389/fendo.2021.772865

**Published:** 2021-11-19

**Authors:** Lingli Xie, Ziying Zhang, Qian Wang, Yangwen Chen, Dexue Lu, Weihua Wu

**Affiliations:** Department of Endocrinology, The 3rd Affiliated Hospital of Shenzhen University, Shenzhen, China

**Keywords:** diabetes mellitus, ACE2 (angiotensin converting enzyme 2), COVID - 19, SARS- CoV-2, therapeutic management, receptor

## Abstract

The potential relationship between diabetes and COVID-19 has been evaluated. However, new knowledge is rapidly emerging. In this study, we systematically reviewed the relationship between viral cell surface receptors (ACE2, AXL, CD147, DC-SIGN, L-SIGN and DPP4) and SARS-CoV-2 infection risk, and emphasized the implications of ACE2 on SARS-CoV-2 infection and COVID-19 pathogenesis. Besides, we updated on the two-way interactions between diabetes and COVID-19, as well as the treatment options for COVID-19 comorbid patients from the perspective of ACE2. The efficacies of various clinical chemotherapeutic options, including anti-diabetic drugs, renin-angiotensin-aldosterone system inhibitors, lipid-lowering drugs, anticoagulants, and glucocorticoids for COVID-19 positive diabetic patients were discussed. Moreover, we reviewed the significance of two different forms of ACE2 (mACE2 and sACE2) and gender on COVID-19 susceptibility and severity. This review summarizes COVID-19 pathophysiology and the best strategies for clinical management of diabetes patients with COVID-19.

## Introduction

The outbreak of the novel coronavirus disease (COVID-19) in 2019 posed a serious and continuing challenge to the global public health system. This disease is caused by a novel sense, single-stranded, enveloped RNA β-coronavirus referred to as the Severe Acute Respiratory Syndrome coronavirus 2 (SARS-CoV-2) (1). As of 31^st^ August 2021, the World Health Organization had reported a total of 216867420 globally diagnosed COVID-19 cases, including 4507837 COVID-19 associated mortalities. The pathophysiology of COVID-19 interacts with that of diabetes, which is a chronic disease. SARS-CoV-2 infection can lead to a new-onset of diabetes and severe metabolic complications in previous diabetes, including diabetic ketoacidosis (DKA) and hyperosmolar hyperglycemic state (HHS). Besides, diabetes is associated with an increased risk of severe COVID-19. Therefore, understanding the clinical processes of SARS-CoV-2 infections and therapeutic efficacies of commonly used drugs in diabetic patients is key in COVID-19 management.

SARS-CoV-2 gains entry into target cells by attaching to angiotensin-converting enzyme 2 (ACE2) receptor. During complex virus-host cell fusion processes, the receptor-binding domain (RBD) of SARS-CoV-2 spike glycoprotein (S1) binds ACE2 (2). Then, proximal serine proteases [such as transmembrane serine protease 2 (TMPRSS2)] cleave the SARS-CoV-2 spike protein and ACE2, thereby promoting viral entry (3).

## Biological Characteristics of ACE2 and ACE

**Figure 1:**
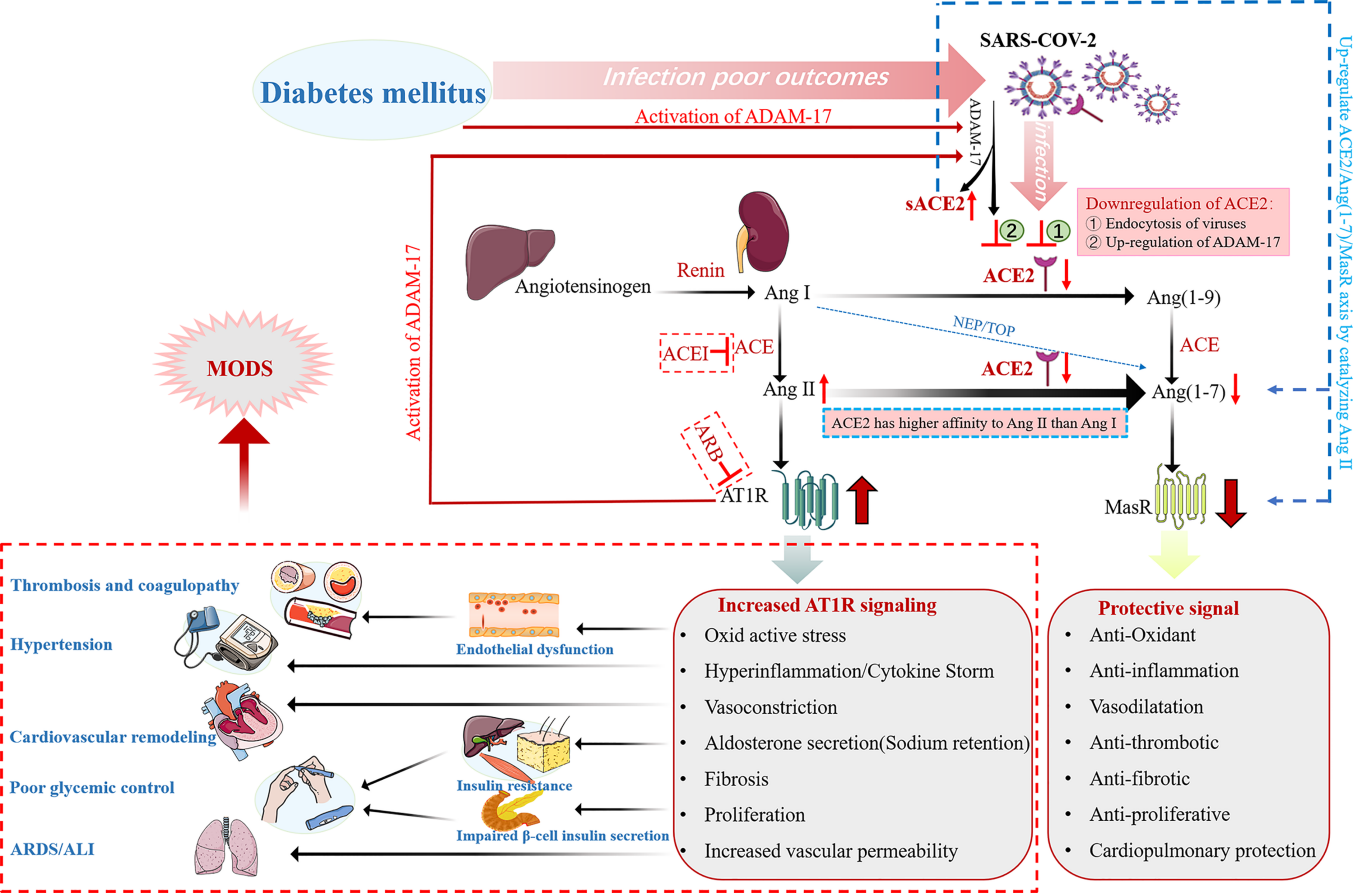
Schematic representation of the alterations of the renin angiotensin aldosterone system (RAAS) in diabetic patients with COVID-19. ADAM-17 is activated in diabetic patients, therefore both DM and SARS-CoV-2 infection downregulate membrane-bound ACE2 in diabetes mellitus patients with COVID-19. ACE2 has a higher affinity for Ang II than Ang I. Therefore, down-regulation of ACE2 leads to Ang II accumulation and decreased Ang (1-7) levels. In turn, accumulation of Ang II activates ADAM-17, resulting in increased sACE2 shedding and a further decrease in membrane-bound ACE2. Thus, the protective signal mediated by ACE2/Ang (1-7) axis is down-regulated, and the detrimental signal mediated by Ang II/AT1R axis is up-regulated, resulting in multiple organ damage effects and even multiple organ failure.

The two enzymes of the renin-angiotensin system (RAS), angiotensin-converting enzyme (ACE) and ACE2, have about 60% homology and play opposite roles. In RAS, renin cleaves angiotensinogen into angiotensin I (Ang I), which is then converted into angiotensin II (Ang II) by ACE. Ang II acts on type 1 angiotensin receptor (AT1R), which increases blood pressure levels by inducing vasoconstriction and by increasing renal reabsorption of sodium as well as water. Moreover, Ang II promotes oxidative stress, inflammation and fibrosis (4). In addition to causing pulmonary edema and lung injury, it can also lead to insulin resistance, endothelial dysfunction and proteinuria. These effects of ACE-Ang II are directly opposite to those of ACE2-Ang- (1-7) signal transduction. ACE2 converts Ang II to Ang 1-7, which acts on Mas receptors and lowers blood pressure levels by mediating vasodilation, promoting renal sodium and water excretion. It suppresses inflammatory response and oxidative stress levels by producing nitric oxide (5). It also has anti-fibrosis, anti-proliferation and anti-thrombosis effects. Therefore, the classical ACE-Ang II-AT1R regulatory axis and ACE2-Ang1-7-MAS anti-regulatory axis can maintain homeostasis *in vivo* **(** **Figure 1** **).**

ACE2 is essential in regulation of lung homeostasis and lung injury prevention. After SARS-CoV-2 binds ACE2 receptors on the surface of alveolar epithelial cells, expressions of ACE2 in alveolar epithelial cells are down-regulated by internalization, shedding, viral replication and other mechanisms (6). Subsequently, elevated Ang II levels trigger inflammatory responses, leading to neutrophil, macrophage and fibrin exudation, thereby aggravating vascular permeability and pulmonary edema. These effects lead to the loss of pulmonary ventilation, persistent oxygenation difficulties, and increased risk of acute respiratory distress syndrome (ARDS) (7). Therefore, during SARS-CoV-2 pathogenesis, ACE2 is involved in viral entry and in lung protection against injury.

## Main Receptor ACE2

There are two forms of ACE2: structural transmembrane protein ACE2 (mACE2) with the extracellular domain, which can act as the receptor for SARS-CoV-2 spike protein, while soluble form represents cyclic ACE2 (sACE2). Under normal circumstances, the functions of ACE2 in the lungs are limited, however, they may be up-regulated in some clinical conditions. Importantly, plasma levels of ACE2 (sACE2) cannot be used as reliable indicators for complete membrane-bound ACE2 (mACE2) activity. This is partly due to ACE2 shedding from the membrane, which seems to be endogenously regulated. In addition to expression levels, biological roles of ACE2 may vary among tissues as well as clinical status.

**Figure 2:**
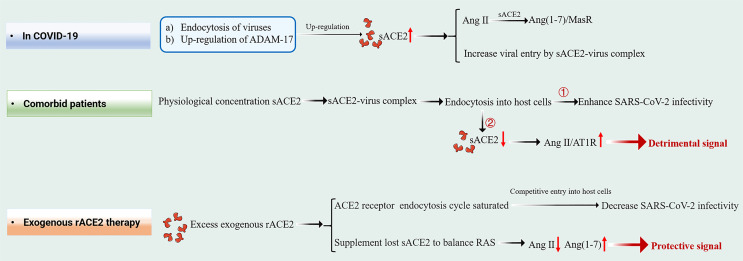
Schematic diagram of the effect of sACE2 in circulation under three different states.

mACE2 is highly expressed in the heart, airway, kidney and liver tissues, while sACE2 is produced by mACE2 shedding in response to inflammatory signals and diseases. A-disintegrin and metalloproteinase (ADAM) 17 cleaves mACE2 to release soluble ACE2 into plasma. Under elevated Ang II conditions, the extracellular catalytic domain of mACE2 is released by ADAM-17 as a feedback mechanism. sACE2, which retains enzyme activity and complete SARS-CoV-2 interaction sites, allows easy access to Ang II to counteract its effects and to bind extracellular floating viral S proteins in a manner similar to mACE2 **(** **Figure 2** **)** (8–10). However, the precise roles of sACE2 during the course of COVID-19 infection are still unclear.

### Membrane Binding Form mACE2

As a COVID-19 receptor, differential expressions of mACE2 may lead to different physiological responses to SARS-CoV-2 infection. Both SARS-CoV-1 and SARS-CoV-2 gain entry into lung cells by binding mACE2. After SARS-CoV-1 infection, ADAM-17 induces mACE2 shedding and enhances the secretion of tumor necrosis factor by cells (11), resulting in elevated Ang II levels and imbalanced RAS signaling (12). In COVID-19, Ang II levels are correlated with lung injury. Therefore, SARS-CoV-2 infected individuals with previous mACE2 deficiencies may be highly predisposed to severe mACE2 deficiency in the lungs, which may increase the risk of acute lung injury and death (13).

The elderly, male gender, complications associated with hypertension, cardiovascular disease, obesity and diabetes mellitus have been identified as important predisposing factors for the development of severe COVID-19 and COVID-19-associated mortality (14–16). Even though the underlying mechanisms of these associations have not been clearly established, the increased risk may be attributed to the relative lack of mACE2 and the corresponding increased expressions of sACE2. mACE2 deficiencies are associated with old age, male gender, cardiovascular disease and diabetes mellitus (13, 17–19). Meanwhile, sACE2 levels have been found to be elevated in elderly individuals, male gender, as well as during cardiovascular and inflammatory diseases (20, 21).

### Soluble Form sACE2

#### In COVID-19

Lung inflammation during COVID-19 is coordinated by Ang II and sACE2 levels. SARS-CoV-2 gains entry into cells by binding mACE2, whose level reduces while that of Ang II, as an early response to SARS-CoV-2 infection, reaches its peak, thereby triggering anti-viral inflammatory responses and neutrophil infiltration into lungs. However, sACE2 levels in the lungs begin to increase with viral entry. At its peak, Ang II increases ADAM-17 levels through AT1R activation, resulting in elevated sACE2 levels, which facilitates the Ang 1-7/MasR pathway, regulates inflammation and prevents further tissue injury (19, 22, 23).

**Figure 3:**
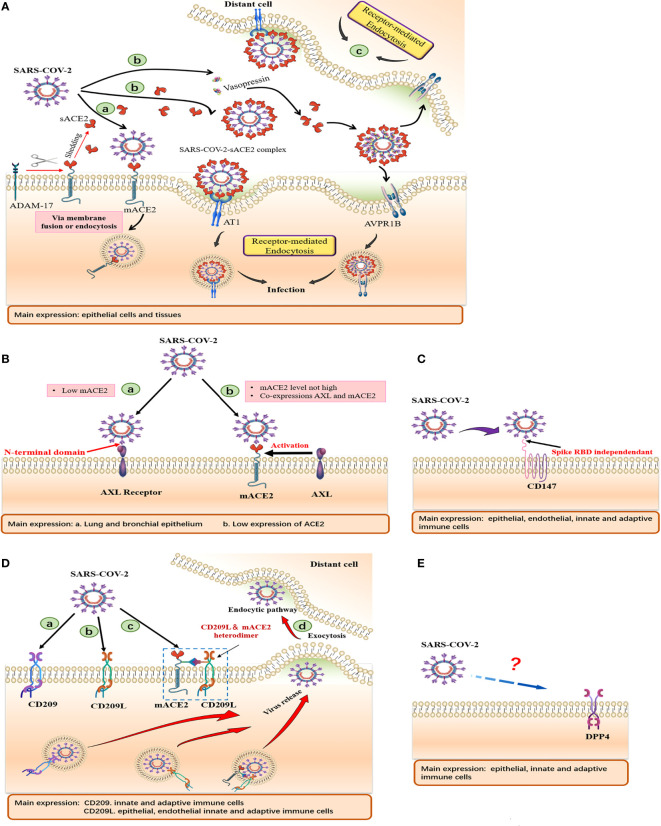
Schematic of cell surface receptors mediated entry of SARS-CoV-2. Summary of tissue and cellular expressions, and models of **(A)** ACE2, **(B)** AXL, **(C)** CD147, **(D)** CD209, CD209L and **(E)** CD26. **(A) (a)** Virus gains entry into host cells *via* mACE2. **(b)** SARS-CoV-2 binds sACE2 to form an sACE2-virus complex, which subsequently enters host cells through AT1 and AVPR1B receptors mediated endocytosis. **(B) (a)** AXL acts as a membrane receptor and its NTD binds SARS-CoV-2 to mediate viral entry. **(b)** AXL, as a host factor facilitating viral entry, cooperates with ACE2 to promote viral entry at low ACE2 expression levels. **(D) (a, b).** CD209 and CD 209L acts as membrane receptors that mediate viral entry. **(c)** CD209L can form heterodimerization complexes with ACE2 to mediate viral entry. **(d)** Cells expressing DC-SIGN and L-SIGN can transfer SARS-CoV-2 to susceptible target cells, release virus through exocytosis and mediate distant cell infection.

The transmission of SARS-CoV-2 between cells has been reported to occur *via* macropinocytosis, an endocytosis process mediated by receptor-independent filamentous pseudopodia. In this case, the virus-sACE2 complex may enter host cells through endocytosis (24). *In vitro *studies have shown that endogenous sACE2 interacts with SARS-CoV-2 spike proteins in the extracellular compartment. The resulting sACE2-S complex binds AT1 surface receptors to enter cells *via* receptor-mediated endocytosis (25). In addition, the S protein of SARS-CoV-2 interacts with vasopressin to form a sACE2-S-vasopressin complex, which facilitates cellular entry through another vasopressin receptor AVPR1B (25). These new mechanisms allow the virus to enter tissues that poorly express mACE2 **(** **Figure 3A** **)**. This explains the result that cells from various organs may be susceptible to SARS-CoV-2 after treatment with recombinant ACE2 (rACE2) (25). A study by Haga et al. (11) showed that cells may be sensitive to SARS-CoV by inducing ADAM17 activity to increase the sACE2 production. The authors reported that SARS-CoV S induced ADAM17 activity, resulting in increased ACE2 shedding, which was positively correlated with SARS-CoV infection.

The virus-sACE2 complex, which is formed by sACE2 binding the SARS-CoV-2 S protein in the extracellular compartment, may have two major adverse effects **(** **Figure 3A**). i. The sACE2 coating on the virus does not interfere with host immune cells, making it easy to spread from the primary site of infection to distant organs to avoid immune attacks, especially in patients with complications, which mediates multiple organ failure. ii. This complex can be internalized by endocytosis. With a large number of sACE2 bound to the complex being internalized into host cells, the rapid decrease in mACE2 and extracellular sACE2 levels as well as the accumulation of Ang II may lead to a serious imbalance in host RAS homeostasis and severe COVID-19.

#### In Exogenous rACE2 Therapy and Comorbid Patients

Kornilov et al. reported that plasma sACE2 levels are higher in men than in women (26), consistent with Sama et al. (20). As sACE2 levels increase with age, adult males have higher sACE2 levels than adult females and children (21). sACE2 levels are elevated in individuals with higher body mass indices (BMI) and metabolic syndromes while the correlation between sACE2 levels and metabolic syndromes is stronger in males (26).

In a global study involving 10 753 participants, elevated plasma ACE2 levels were correlated with an increased risk of major cardiovascular events (including death, myocardial infarction, stroke, heart failure and diabetes) (27). A recent large-scale study reported that sACE2 levels in patients with severe COVID-19 were higher than in patients with non-severe COVID-19 (28). However, as a potential risk marker for severe COVID-19, the roles of sACE2 have not been fully evaluated. Therapeutic human recombinant soluble ACE2 (hrsACE2) or engineered sACE2 have been shown to reduce Ang II levels, substantially suppress the expressions of key cytokines associated with COVID-19 pathogenesis and prevent viral entry in patients receiving such treatments and in cell experiments (29, 30). Plasma ACE2 (sACE2) needs special considerations. We cannot yet adequately consent with the claim that it decreases viral entry.

##### Exogenous rACE2 Therapy

With regards to inhibition of viral entry, therapeutic sACE2 exhibited dual effects in SARS-CoV-2 infection **(** **Figure 2** **)**. In *in vitro* models, very high concentrations of rACE2 [10-200 μg/mL of ACE2, which are much higher than its physiological concentration (20, 31)] inhibited SARS-CoV-2 infection. These findings are consistent with the results of Yeung et al. who showed that 25 and 100 μg/mL rACE2 can inhibit SARS-CoV-2 infection. We postulated that excess administration of exogenous rACE2 (therapeutic sACE2, at μg/mL level) might saturate the endocytosis cycle of ACE2 receptor, compete with SARS-CoV-2- ACE2 complex to enter host cells, resulting in reduced infectivity of SARS-CoV-2. In contrast, rACE2 at near-physiological concentrations (that is, ηg/mL levels) can enhance SARS-CoV-2 infectivity (25). Interestingly, a similar phenomenon was reported in a recent study (29), which showed that after the administration of rACE2, the viral loads were detected substantially and stably increased in nasopharynx swabs (from ∼10^4^ copies/mL at day 0 to ∼10^5^ copies/mL at day 5) and tracheal aspirates (from ∼10^3^ copies/mL at day 0 to ∼10^5^ copies/mL at day 2), respectively. Although patients eventually recovered after development of neutralizing antibodies, this *in vivo* data and results from the study by Yeung et al. suggest that the role of sACE2 in SARS-CoV-2 infection should be carefully considered.

Natural and therapeutic sACE2 retain Ang II catalytic activities, in addition to binding viral RBD. Additional infusions of sACE2 supplements lost sACE2 and is important in temporarily balancing RAS during exogenous rACE2 therapy. Continuous transformation of Ang II into Ang1-7 protects Ang II-mediated severe pathological processes. Therefore, exogenous hrsACE2 treatment suppresses excess RAS activation and the increase in Ang II concentrations, thereby reducing damage to multiple organs, including the lungs, kidneys and heart (12, 32, 33).

##### Patients With Complications

Although comorbid patients have more sACE2, the opposite phenomenon is observed, and COVID-19 patients with comorbidities suffer more serious consequences. We believe that the half-life of natural sACE2 in circulation is short and it occurs in very low levels. It gains entry into host cells in form of virus-sACE2 complex, which may be the reason as to why comorbid patients fail to obtain protection. We propose a viral entry mechanism for comorbid patients. SARS-CoV-2 in the extracellular compartment can bind a large number of sACE2 molecules to form sACE2-viral complexes, and sACE2 levels in circulation decrease dramatically after endocytosis. The resulting rapid rise in Ang II levels may lead to cytokine storms and other pathological complications through the Ang II-AT1R axis, aggravating disease severity in comorbid patients **(** **Figure 2** **)**.

As for the effect of soluble ACE2 in SARS-CoV-2 infection, these considerations need to be examined and confirmed experimentally to clarify the precise role of sACE2 *in vitro* and *in vivo*. Biological mechanisms of elevated sACE2 levels are still an active research field, which is likely to be a common result of complex interactions between impaired cell expressions, enzymatic digestion and impaired plasma clearance. However, our biological understanding of the roles of sACE2 is limited and we cannot infer their functions at the biological tissue level.

## Other Membrane Receptors That Mediate SARS-CoV-2 Infection

ACE2 is expressed in different epithelial cells, including those of the lungs, kidneys, intestines, heart, brain neurons, immune cells, pancreas and blood vessels. Its tissue distribution is organ-specific. Elevated expressions have been found in the kidneys, cardiovascular and gastrointestinal systems (17, 34, 35). Expression levels of ACE2 in the lungs are very low, and they are only expressed in a small part of lung epithelial cells (36), moreover, they may even be undetectable in endothelial cells of lung tissues. ACE2 is rarely expressed in immune cells, but they can be infected by SARS-CoV-2, suggesting that SARS-CoV-2 may enter and infect some human cells by attaching to other receptors, or to combinations of multiple receptors and/or enhancers. These may be the key to infection of tissues with low or without ACE2 expression.

## Receptor: AXL

The TAM phosphatidylserine receptor family, AXL, is expressed in almost all human organs, especially in the lungs, bronchial epithelial tissues and cells, where AXL levels are much higher than those of ACE2. ACE2 is rarely expressed in the lungs and trachea (37).

Overexpressed AXL in AXL/ACE2 double KO HEK293T cells promote viral infection, reaching levels that are comparable to those of HEK293T cells overexpressing AXL. AXL down-regulation in ACE2-KO H1299 cells was shown to significantly reduce SARS-CoV-2 pseudotyped infections (38). Human recombinant soluble AXL (rather than ACE2) prevents SARS-CoV-2 infection in cells with elevated AXL expressions. Besides, expression levels of AXL are closely correlated with SARS-CoV-2 S protein levels in bronchoalveolar lavage fluid cells from COVID-19 patients (38). These findings suggest that AXL may be a novel host receptor that mediates SARS-CoV-2 entry and infection. Since AXL is not co-expressed with TMPRSS2 or ACE2 in human lung and tracheal cells, sACE2 fails to prevent cells overexpressing AXL from SARS-CoV-2 infection, and vice versa, implying that AXL is independent of ACE2-mediated viral entry **(** **Figure 3B** **)**.

AXL and SARS-CoV-2 S are mainly co-localized in the cell membrane. Contrary to the ACE2 receptor, AXL directly interacts with the SARS-CoV-2 spike protein N-terminal domain (NTD) rather than RBD (38). Evidence obtained by measuring viral RNA levels adsorbed on cell surfaces and internalized viral RNA in cells revealed that AXL enhances SARS-CoV-2 infection by promoting viral adsorption.

These data are in contrast to the conclusions of another report, which points out that AXL does not interact with SARS-CoV-2 spike proteins, and neither does it mediate SARS-CoV-2 entry unilaterally, however, it is a host factor promoting SARS-CoV-2 entry (39). Under synergistic effects of low-level ACE2, AXL interacted with viral particle-related phosphatidylserine (PS) to enhance SARS-CoV-2 infection, while the effects of AXL were no longer observed at high ACE2 levels (39). Since expression levels of ACE2 in the lungs are low, AXL may be highly correlated with SARS-CoV-2 infections.

## Receptor: CD147

CD147, also known as basigin (encoded by BSG), is a transmembrane receptor. Wang et al. (40) for the first time, reported on the direct interactions between CD147 and SARS-CoV-2 spike proteins, which mediate host cell infection. SARS-CoV-2 viral loads were detected in the lungs of hCD147 mice infected with SARS-CoV-2, but not in the lungs of wild-type mice infected with SARS-CoV-2. In another study, SARS-CoV-2 pseudovirus was shown to infect ACE2 deficient T cells in a dose-dependent manner, and this infection was specifically inhibited by a humanized anti-CD147 antibody (Meplazumab), suggesting that overexpression of CD147 promotes viral infection (40). This result provides a possible explanation for lymphocyte reduction in COVID-19 patients. In addition, Meplazumab can effectively promote the rehabilitation of patients with SARS-CoV-2 pneumonia (41).

mAb, which targets spike RBD, does not prevent viral entry in SW480 and A549 cells (these cells express CD147 but have a low abundance of hACE2). However, anti-CD147 mAb inhibits viral infections in A549 cells (42), indicating that RBD does not interact with CD147 and is not directly involved in CD147-mediated infections in these cells. Besides, CD147 knockdown in Calu-3 cells expressing high levels of hACE2 did not affect susceptibility to SARS-CoV-2 (42), indicating that CD147 seems to act as an alternative receptor in cells without or with a low abundance of hACE2.

Urszula et al. reported elevated CD147 levels in epithelial tissues, congenital and adaptive immune cells, which acted as SARS-CoV-2 receptors (43). Potentially, these cells can be infected in the lungs or can carry SARS-CoV-2 from infected cells through CD147 to participate in local and systemic transmissions of the virus, thereby enhancing the exaggerated immune responses (44).

Interestingly, immunofluorescence assay did not show co-localizations of CD147 and ACE2 in lung tissues of COVID-19 patients, and neither did it show interactions in detected cells **(** **Figure 3C** **)**. Furthermore, expressions of CD147 and ACE2 in a single lung cell were found to be completely independent, indicating that CD147 and ACE2 may be two complementary receptors mediating SARS-CoV-2 infection (40).

Hyperglycemia and RAGE activation can upregulate expressions of the CD147 glycoprotein while advanced glycation end products (AGEs) have been shown to significantly elevate the expression levels of the CD147 protein through RAGE-dependent mechanisms (45). Due to elevated AGEs expressions in diabetic patients, corresponding CD147 protein expression levels increased, which may increase the accessibility of SARS-CoV-2 to diabetic tissues. Relative to healthy controls, plasma CD147 levels have been reported to be elevated in diabetic patients and can be used to predict 10-year mortality rates (46). CD147 overexpressing in host cells may promote more viral entry, which may partially explain the high mortality rate in diabetic patients with COVID-19.

## Receptors: DC-SIGN and L-SIGN

Endothelial cells lacking ACE2 receptors can also be infected by SARS-CoV-2. Suppression of CD209L expressions or functions in endothelial cells by soluble CD209L or shRNA inhibited SARS-CoV-2 entry. In addition, ectopic expressions of CD209 and CD209L in HEK-293 cells enhanced SARS-CoV-2 entry (47). Amraei et al. reported that the RBD of SARS-CoV-2 S protein binds CD209L and CD209 to mediate viral entry (47). Kondo et al. documented that L-SIGN mediates viral entry by interacting with high-mannose–type N-glycans on SARS-CoV-2 spike proteins. Blockade of L-SIGN functions can significantly reduce SARS-CoV-2 infections (48). These findings indicate that in addition to ACE2, CD209L/L-SIGN and CD209/DC-SIGN can act as substitute receptors for mediating SARS-CoV-2 entry into host cells **(** **Figure 3D** **)**.

CD209L is highly expressed in renal proximal epithelial cells, alveolar type II epithelial cells, as well as in endothelial cells of blood vessels, lungs, liver and lymph nodes (47, 49, 50), CD209 is mainly expressed in tissue-resident macrophages, dendritic cells and B cells (51, 52). These findings suggest that SARS-CoV-2 can invade these cell types by binding CD209L and CD209. Through single-cell RNA sequencing analysis, Chao et al. found that CD209/DC-SIGN is highly expressed in innate immune cells and lymphoid organs (53). They also observed that DC-SIGN can directly bind the SARS-CoV-2 S glycoprotein with a picomolar affinity, trigger S internalization by 3T3DC-SIGN + cells, which might provide a pathway for SARS-CoV-2 to enter macrophages and dendritic cells. These newly discovered SARS-CoV-2 receptors on innate immune cells may aggravate cytokine release syndrome and severe pathological inflammation.

In addition, the vascular system is the main attack site of SARS-CoV-2. Patients with COVID-19 exhibit endothelial cell injuries (namely endodermis), angiogenesis changes and extensive microvascular thrombosis (54, 55). SARS-CoV-2 gains entry into endothelial cells using CD209L as the receptor. This leads to suppressions of CD209L functions in endothelial cells and alterations in angiogenesis as well as endothelial cell damage (47).

CD209L and CD209 have been shown to directly physically interact with the SARS-CoV-2 S-RBD protein. Consistent with the findings of Amraei et al., van Kooyk et al. reported that CD209L binds the S protein of SARS-CoV-2 in an ACE2-independent manner (56). It has also been reported that the S protein of SARS-CoV-2 binds CD209 in a glycosylation-dependent manner (53). Co-immunoprecipitation assays showed that CD209L can form heterodimerized complexes with ACE2, which may play an important role in infection of cell types expressing both CD209L and ACE2 (47). These findings indicate that CD209L and ACE2 are co-receptors for SARS-CoV-2 infections. Taken together, CD209L promotes SARS-CoV-2 entry into host cells in ACE2-dependent and non-dependent ways.

Cells expressing DC-SIGN and L-SIGN can transfer SARS-CoV to susceptible target cells (57, 58). Therefore, it is postulated that SARS-CoV-2 binds CD209L and CD209 receptors on innate immune cells, which then transfer and redistribute them to target tissues, where they cause more damage after viral internalization (59). This hypothesis provides a possible explanation for how SARS-CoV-2 spreads to extrapulmonary tissues in the host.

## Receptor: DPP4

Another potential receptor that may explain the association between COVID-19 and diabetes involves the dipeptidyl peptidase-4 (DPP4 or CD26) enzyme **(** **Figure 3E** **)**, which is widely expressed in many tissues (such as kidneys, lungs, intestines and immune cells) and plays a key role in inflammation as well as glucose homeostasis. DPP4, a common pharmacological target of type 2 diabetes (T2DM) and a functional coronavirus receptor, can enhance sensitivity to coronavirus infections. In MERS-CoV infections, MERS-CoV S glycoprotein RBD binds human receptor dipeptidyl peptidase 4 (hDPP4) to mediate viral entry (60). A large proportion of COVID-19 cases are accompanied by different severity of neurological symptoms. Madeline et al. reported that cortical astrocytes, which had lowest ACE2 levels and elevated DPP4 levels were significantly and mainly infected. Inhibition of DPP4 can attenuate viral infection and decrease the expression levels of the cell stress marker, ARCN1. DPP4 mediates SARS-CoV-2 tropism to human astrocytes, resulting in reactive glial hyperplasia injury (61). Vankadari et al. predicted the interactions between DPP4/CD26 and the S1 domain of SARS-CoV-2 spike protein, thereby elucidating on the complex docking model of SARS-CoV-2 spike glycoprotein and DPP4 (62). This implies that the complementary virus-host interaction was in addition to main interactions between ACE2 and the S protein.

Indeed, Tai et al. (63) reported that the RBD of SARS-CoV-2 can bind 293 T cells expressing human ACE2, but cannot bind 293 T cells expressing human DPP4. Flow cytometry analysis further showed that the binding of SARS-CoV-2 RBD to 293 T cells can be significantly blocked by the sACE2 protein, but not by sDPP4. Furthermore, the SARS-CoV-2 RBD cannot prevent MERS-CoV pseudovirus from entering 293T cells expressing hDPP4 (63). Hence, in the absence of experimental verification, bioinformatics data should be interpreted with caution. Although it is necessary to clarify the direct relationship between DPP4 and SARS-CoV-2 infection, evidence suggests that the DPP4 inhibitor (DPP-4i) can regulate inflammation and exert antifibrotic activities. These properties may have potential applications in preventing progression to an over-inflammatory state associated with severe COVID-19.

## Diabetes Increases COVID-19 Severity

**Figure 4:**
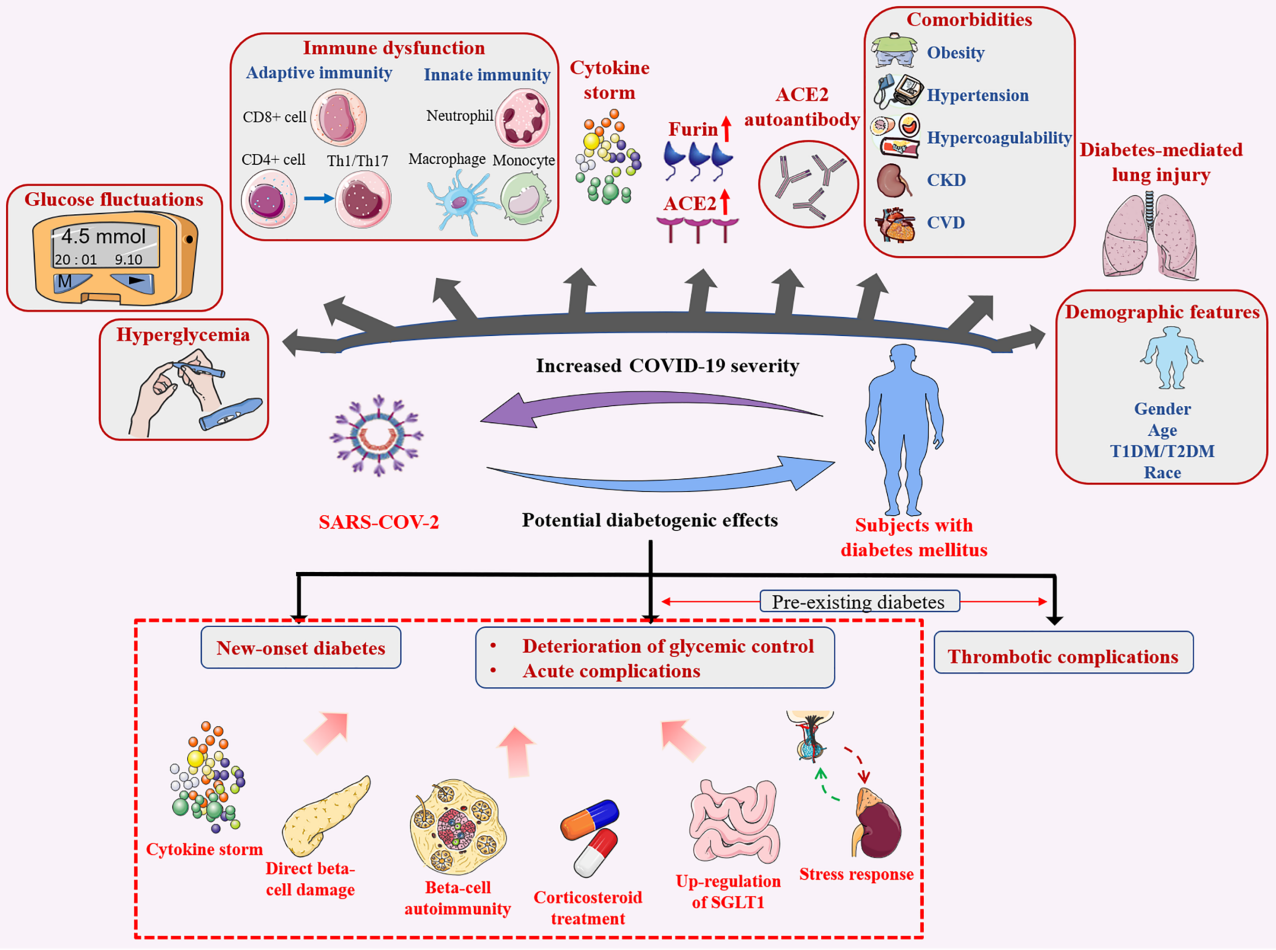
Bidirectional effects between diabetes mellitus and SARS-CoV-2 infection. Diabetes mellitus contributes to poor infection outcomes, SARS-CoV-2 infection has potential diabetogenic effects.

Diabetes does not increase the risk for SARS-CoV-2 infection, but significantly increases COVID-19 severity and associated mortality rates. These outcomes are mediated by hyperglycemia and blood glucose fluctuations, expressions of furin proteins and ACE2 receptor, ACE2 autoantibodies production, imbalances in immune and inflammatory pathways, diabetes-related complications as well as lung injury in diabetes. In addition, demographic features (e.g., age, gender) affect COVID-19 prognosis **(** **Figure 4** **)**.

### Hyperglycemia and Glucose Fluctuations

Baseline blood glucose and continuous glycemic control in hospitalized patients, rather than T2DM itself seem to be associated with COVID-19 progression (64). Fasting blood glucose levels at admission, rather than previously diagnosed diabetes was found to be an independent predictor for severe illness (65), death (66), or adverse outcomes (67) in hospitalized COVID-19 patients.

Elevated plasma glucose levels and glycolysis in human monocytes enhance and sustain SARS-CoV-2 replication (68) while elevated plasma glucose levels promote viral replication (69). These findings imply that hyperglycemia may inhibit antiviral immune responses (70), which may explain the prolonged rehabilitation time among diabetic patients with COVID-19.

In addition, SARS-CoV2 causes acute glucose fluctuations in diabetic patients, and glycemic variability may exert negative effects on patient rehabilitation (71, 72). Fluctuations in blood glucose levels are also true for diabetic patients. Therefore, in addition to long-term glycemic control (73), acute hyperglycemia may play a key role in worsening COVID-19 prognosis (74).

### Immune Dysfunction and Cytokine Storm

Congenital and adaptive immunity, inflammation and coagulation abnormalities are significantly more in diabetic COVID-19 patients than in non-diabetic COVID-19 patients (75), which promotes COVID-19 progression, independent of other complications (76), and are associated with blood glucose levels (77). Nevertheless, delayed immune responses, the disproportionate number of immune cells and high levels of inflammation in diabetic patients may exacerbate them to ‘cytokine storm’, which seems to be directly correlated with multiple organ dysfunction, death and COVID-19 severity (78–80).

### Expression of ACE2 and Furin in Diabetes

SARS-CoV-2 binds ACE2, which is subsequently cleaved by proteases, such as TMPRSS2 and furin, leading to internalization of virosome complexes (81). One of the mechanisms through which diabetes increases the risk of serious infection may be attributed to increased expressions of ACE2 receptor and furin, which may promote the entry and replication of SARS-CoV-2 (81, 82). Due to the increase in efficient virus entry, viral load increases, resulting in a poor prognosis of COVID-19. An elevated serum furin level is a marker of diabetes progression, and is correlated with metabolic abnormalities as well as an increased risk of premature diabetes-associated death (83). In diabetic rodent models, ACE2 expressions were also reported to be elevated in the lungs, kidneys, heart and pancreas (84, 85). The relationship between diabetes and ACE2 expression in human lungs has not been fully established. Rao et al. (86) conducted a full-phenotypic Mendel randomization study and found that diabetes has a causal relationship with elevated ACE2 expression levels in the lungs, which may promote susceptibility to severe complications of SARS-CoV-2 infections. While some evidence shows that ACE2 levels in renal tissues of diabetic patients are down-regulated (87, 88), suggesting that ACE2 expressions in diabetic patients vary among tissues. ACE2 has been found to be expressed in multiple organs - a possible reason for multiple organ failure in some COVID-19 patients.

### ACE2 Autoantibodies

ACE2 autoantibodies were found in SARS-CoV-2-infected patients (89–91). Once the body generates antibodies against SARS-CoV-2 RBD, antibodies that recognize and inhibit self ACE2 may also be produced. Through this mechanism, the virus triggers autoimmune diseases (92). Therefore, ACE2 autoantibodies may be considered anti-idiotypic antibodies. Autoantibodies against ACE2 (AA-ACE2) can be used to reflect the severity of COVID-19. The AA-ACE2 level in patients with COVID-19 was significantly higher than that in the control group without infection. Of note, AA-ACE2 levels in moderate and severe COVID-19 patients were significantly higher than in mild patients (91). Similarly, Wang et al. found that AA-ACE2 levels were higher in COVID-19 patients, especially in severe patients, than in healthy controls. The AA-ACE2 can alter the course of COVID-19 by disrupting immune response to SARS-CoV-2 and tissue homeostasis (93).

The AA-ACE2 level in patients with diabetes (Median 16.630 [IQR 10.480–27.356] U/mL) was significantly higher than that in non-diabetic patients (Median 7.957 [IQR 4.339–19.715] U/mL) (91). Notably, there were differences in AA-ACE2 levels among patients with the same severity level, this may be caused by individual differences in immune response as well as consequences of previous complications (such as diabetes). The relationship between AA-ACE2 and disease severity is also applicable for COVID-19 patients with diabetes (91).

Plasma from patients with ACE2 autoantibodies inhibited exogenous ACE2 activity whereas that obtained from patients without ACE2 autoantibodies did not (91). This inhibition may reduce the activity of membrane-bound ACE2 and soluble ACE2 (89). In COVID-19, circulating ACE2 levels were increased as a function of virus-induced ACE2 shedding. The elevated ACE2-SARS-CoV-2 complex level in circulation may enhance the production of AA-ACE2. High levels of AA-ACE2 may further decrease the activity of membrane-bound ACE2 in lung and other tissues, which is likely to increase Ang II levels and immune system activation, thereby transforming the RAS balance to the pro-inflammatory axis. Hence, AA-ACE2 (by reducing the activity of transmembrane ACE2 and transformation of Ang II to Ang 1-7) increased the activity of AT1 receptor, enhanced pro-inflammatory response, and ACE2 shedding (94, 95), thereby aggravating the severity of COVID-19.

### Diabetes-Related Complications

In addition to the negative impact of hyperglycemia on COVID-19, diabetes-related chronic complications such as obesity, hypertension, coronary arterial disease and chronic kidney disease may further deteriorate COVID-19 progression (96–103). Diabetes and related complications are also associated with increased fibrinolytic enzyme expression levels. The fibrinolytic enzyme is a protease that can cleave the S protein of SARS-CoV2, which is beneficial for the virus to combine with ACE2 and enter cells to increase the virulence and infectivity of SARS-CoV-2 (104). Furthermore, fibrinolytic enzyme-associated degradation of blood fiber proteins leads to elevated D-dimer levels and other degradation products of blood fiber proteins, which is a feature of severe disease (104). Complications of diabetic patients are independent of diabetes itself, and they are associated with poor COVID-19 prognosis (73, 105).

### Lung Injury in Diabetes

Diabetes is associated with physiological and structural abnormalities in lung tissues and impaired lung functions (106). Forced vital capacity is reportedly low in T1DM patients, which is thought to be associated with poor glycemic control (107). Philips et al. (108) found that in animal models, diabetes was correlated with changes in lung structure, increased pulmonary vascular permeability and contributed to alveolar epithelial collapse. Therefore, given the impaired respiratory function in diabetic patients and the tendency of SARS-CoV-2 to attack lung tissue cells, it may aggravate COVID-19 pulmonary complications.

### Demographic Features

#### Gender

Global Health 50% data tracker (109) provides data on COVID-19 confirmed cases and deaths from countries around the world according to gender. As of April 2021, the number of male and female COVID-19 confirmed cases was generally comparable, however, the mortality rate among male patients was found to be high. Two reports from the United States (83, 110) and one report from Italy (111) showed that the number of COVID-19-associated deaths in males is higher than in females, however, the incidence of SARS-CoV-2 infection in females is higher. These studies did not classify infections based on age. Risk assessment data of COVID-19 confirmed cases from the European Union/European Economic Zone (EU/EEA) countries and the United Kingdom (UK) showed that the proportion of males requiring hospitalization, intensive care, respiratory support and death was higher than that of females. And the overall ratio of male to female confirmed cases was 0.9, the proportion of males in infected elderly cases was higher, while the proportion of females in infected young cases was higher (112). Indeed, reports from Switzerland and Germany confirmed increased incidences of COVID-19 among men aged over 60 years (113, 114). Nevertheless, the equal absolute number of cases between men and women may indicate a higher incidence of SARS-CoV-2 infection in young women.

These gender differences may be attributed to variations in expression levels of ACE2 and TMPRSS2, and the influence of hormones on immune responses (115). Based on existing evidence, we only analyzed the differences in expressions of the two forms (mACE2 and sACE2) of the main receptor ACE2, and proposed two mutually exclusive mechanisms for explaining gender susceptibility and infection outcomes for COVID-19.

Poor outcomes among males with SARS-CoV-2 infections were speculated to be mainly related to insufficient mACE2 and elevated sACE2 levels. i. Older age, male gender and higher BMI may enhance mACE2 shedding, leading to relative mACE2 deficiency. Jehpsson et al. (116) reported that renin levels increase with age and from puberty, these levels are elevated among men than in women with a positive correlation with sACE2 levels. In circulation, renin levels and activities are correlated with AngII levels (117). Renin may promote ADAM-17-induced mACE2 shedding by affecting AngII levels. Hence, in COVID-19, increased RAS signals with age may lead to elevated AngII/α integrin and ADAM-17-induced mACE2 shedding. Therefore, potential age and gender differences in RAS signals between children and adults as well as between males and females (118) may theoretically lead to mACE2 deficiency among young men (compared to children and women), which may increase the risk for severe COVID-19. These findings are in tandem with those found in rat models (17). ii. Male gender, smoking, older age, high blood pressure and BMI are associated with higher sACE2 levels in circulation. And sACE2 at near-physiological concentration has been proposed as a potential biomarker for COVID-19 severity and can enhance SARS-CoV-2 infection. Moreover, the sACE2-virus complex can be endocytosed by host cells, leading to a sharp decline in sACE2 levels. The subsequent rapid increase in Ang II levels aggravates disease severity through the Ang II-AT1R axis. iii. Low androgen levels among women may inhibit TMPRSS2 expressions, implying that it may be a further protective factor against COVID-19 (119).

Reasons as to why young women have a higher SARS-CoV-2 infection incidence have not been established. However, we postulate that it could partially be due to elevated ACE2 gene expression levels. Through analysis of the GTEx and other public data in 30 organizations of thousands of individuals, expression levels of the ACE2 gene in young people, especially women, were found to be high, while in men, they were low, and the ACE2 gene expressions further decreased with age and T2DM occurrence (120). The data involving humans and mice (120) further revealed that T2DM and inflammatory cytokines are involved in inhibition of ACE2 expressions. Estrogen significantly increases the expressions of the ACE2 gene, while androgen moderately elevates its expression. Age-associated decrease in sex hormone levels can lead to suppressed ACE2 expressions while elevated ACE2 mRNA levels may increase ACE2 activity. Therefore, as a receptor for SARS-CoV-2, elevated ACE2 gene expressions among women may increase the viral load by increasing viral invasion, thereby increasing the risk of SARS-CoV-2 infection.

However, from the perspective of sACE2, males exhibit higher plasma sACE2 levels (26, 27). sACE2 at near-physiological concentration has the ability to bind SARS-CoV-2 and mediate viral infections. However, the half-life of natural sACE2 in circulation is short and it occurs in very low levels (there is little difference between males and females). Therefore, mACE2 plays a leading role in mediating SARS-CoV-2 infections. Nevertheless, it should be noted that there is evidence for mismatches between mRNA levels of ACE2 and ACE2 activity or protein expression in many tissues (121). Thus, the susceptibility of SARS-CoV-2 cannot be fully explained based on ACE2 mRNA expression pattern alone.

#### Age

The severity of infection significantly increases with age. Inferences from age-specific COVID-19-related mortality data from 45 countries revealed that mortality rates among children aged 5-9 years were the lowest, and there was a strong logarithmic linear relationship between age and mortality risk in individuals aged 30-65 years (122). Another study showed that the death risk for patients aged 80 and above was 12 times that of patients aged 50-59 (123). Age-related immune dysfunction, namely immunosenescence and inflammation, plays an important role in increasing vulnerability to severe COVID-19 outcomes among the elderly (124).

#### T1DM/T2DM

Evidence for COVID-19 in T1DM and T2DM is inconclusive. A large National Health Agency (NHS) study in England evaluated COVID-19 mortalities in T1DM (n = 364) and T2DM (n = 7,434) patients. Multiple ORs of 2.86 (95% CI: 2.58-3.18) and 1.80 (95% CI 1.75-1.86), respectively, were reported (125). These findings are supported by a cohort study based on COVID-19 infection, which reported a higher severity and risk of death in T1DM patients than in T2DM patients  (73). In another prospective cohort study involving all Scottish populations, OR values of COVID-19 risk for T1DM and T2DM patients with fatal or intensive care unit (ICU) treatment were 2.40 (95% CI 1.82, 3.16) and 1.37 (95% CI 1.28, 1.47), respectively (126). In contrast, multicenter French Coronavirus SARS-CoV-2 and Diabetes Outcomes (CORONADO) study did not reveal differences in COVID-19 primary outcomes between T1DM and T2DM patients. However, this study only involved 39 T1DM patients (105). Moreover, Wargny et al. reported that the risk of death in hospitalized T1DM patients with COVID-19 on day 7 was only half of that for T2DM patients (10.6% *vs* 5.4%) (127), this study based on the CORONADO Study. Most of the current findings indicate a higher risk of death in T1DM patients, although other studies are yet to confirm this finding.

#### Race

In addition to the abovementioned factors, the race is also a factor associated with increased COVID-19 severity. Among the three major global ethnic groups (African, Asian, White), expression levels of ACE2 differ. Among East Asians, the most important quantitative expression locus (eQTL) contributing to elevated ACE2 expressions is close to 100%, which is more than 30% higher than that of other ethnic groups (120).

## COVID-19 Potential Diabetogenic Effect

The SARS-CoV-2 infection has a huge impact on diabetes progression. It alters immune system responses and promotes inflammation, which may, in turn, lead to glucose imbalance among patients with previous diabetes and even new-onset diabetes. This is an amplification cycle and forms a vicious cycle **(** **Figure 4** **)**.

### COVID-19 Induced New-Onset Diabetes

Some viruses could cause autoimmune type 1 diabetes in patients with genetic susceptibility after infection, and even fulminant diabetes due to massive damage to islet β cells (128). SARS-CoV-1 can cause secondary hyperglycemia (129). Yang et al. compared SARS patients without T2DM history and steroid treatment with matched healthy compatriots. They found that, during hospitalization, more than 50% of patients suffered from diabetes due to SARS-CoV infection. After three years of recovery from viral infections, only 5% of patients still suffered from diabetes (130). Immunostaining of the ACE2 protein also revealed that it was strongly expressed in the islets, but weakly expressed in exocrine tissues, indicating that SARS-CoV might bind ACE2 receptors in the pancreas, leading to acute diabetes by damaging the islets (130). Similarly, the initial diabetes diagnosis is very common in SARS-COV-2 infections, and is not associated with a previous history of diabetes or with the use of glucocorticoids (131), this newly-occurring hyperglycemia is an independent predictor of death (132). According to a meta-analysis involving eight studies, approximately 14% of COVID-19 patients develop new-onset diabetes after hospitalizations (133). By measuring plasma amylase and lipase levels, Wang et al. found pancreatic injury in 9/52 patients with SARS-CoV-2 associated pneumonia, with 6/9 of them exhibiting a moderate increase in blood glucose levels (134). Another study reported that 17% of severe COVID-19 patients had elevated lipase and amylase levels while 7.5% had focal pancreatic enlargement or pancreatic duct dilatation (135). ACE2 is highly expressed in the liver, endocrine pancreas, adipose tissues, kidneys and in the small intestines (136, 137). Therefore, we postulate that SARS-CoV-2 may bind ACE2 in important organs such as the liver and pancreas, and play a potential role in β-cell damage and insulin resistance, thereby inducing new-onset diabetes or worsening diabetes prognosis.

After recovery from COVID-19, it has not been determined whether blood glucose levels return to normal. Long-term assessment of pancreatic β-cell function is recommended to identify future diabetes mellitus development. The COVID-19-related diabetes global registration system (COVIDIAB project) has been established, which may clarify the course and prognosis of new-onset diabetes in COVID-19.

### Deterioration of Blood Glucose, Severe Metabolic Complications and Thromboembolism Induced by COVID-19

#### Deterioration of Glycemic Control and Acute Complications

In diabetic patients infected with SARS-CoV-2, insulin doses increase while blood glucose levels become difficult to control (76, 131, 138, 139). Zhou et al. (138) retrospectively analyzed capillary blood glucose (BG) levels in 881 diabetic patients with COVID-19. They reported that 69.0% of the patients had unsatisfactory BG levels, while 10.3% had at least one hypoglycemia attack. Hypoglycemia is associated with higher cardiovascular-related mortality in diabetic patients (140). In addition, COVID-19 is associated with severe diabetic metabolic complications, including DKA and HHS (76, 141). Hyperglycemia and/or DKA often occur in T1DM patients after COVID-19 infections (142).

Inflammatory storms, β-cell damage, triggering of beta-cell autoimmunity, SGLT1 dysfunction, elevated stress levels and corticosteroid treatment after SARS-CoV-2 infection may act com-binatorially, which not just induces new-onset diabetes, also aggravates hyperglycemia and abnormal blood glucose fluctuations, even leads to DKA, HHS and hypoglycemia.

##### Inflammatory Storms

The relationship between COVID-19 and hyperglycemia in T2DM patients might reflect metabolic inflammation and excess release of cytokines. In diabetic patients, basal cytokine levels, such as IL1-β and IL-6, are elevated, and they are in a mild chronic inflammatory state, which is further amplified by SARS-CoV-2 infection, thereby promoting the vicious circle of cytokine release and rapid increase in blood glucose levels. Elevated cytokine levels (especially IL-6) cause invasive inflammatory reactions referred to as “cytokine storms”, which result in more extensive multiple organ damage. At the same time, it also alters the AMPK/mTOR signaling pathway in diabetic patients, which may aggravate insulin resistance and development of diabetes-related complications. In addition, subclinical inflammatory responses, especially elevations in both IL-1β and IL-6, have been shown to occur prior to the onset of T2DM (143), which further suggests that COVID-19 may increase the risk of new-onset diabetes.

##### Direct β-Cell Damage

Islet β cell injury is associated with various factors. i. As earlier mentioned, ACE2 is also expressed in islet β cells, SARS-CoV-2 enters islet cells by binding ACE2 receptors, thereby resulting in inflammatory cytokine release, β-cell apoptosis and reduced insulin secretion (30). Similarly, studies by Müller et al. have shown that SARS-CoV-2 infects and replicates in cultured human islet cells, leading to morphological and functional abnormalities (144). ii. Interestingly, ACE2 knockout mice were shown to be more susceptible to β-cell dysfunction (145), which explains why SARS-CoV-2 infection can lead to secondary hyperglycemia in patients without previous diabetes. RAS plays a key role in liver and adipose tissue inflammation, insulin resistance and poor glucose tolerance (146, 147). The unique interactions between SARS-CoV-2 and RAAS may provide another mechanism. Down-regulation of ACE2 after SARS-CoV-2 infection may lead to excess Ang II accumulation. It is worth noting that AT1R exists in pancreatic β and α cells of both mice (148) and human beings (149). Therefore, through ACE-Ang II-AT1R axis, it may reduce blood flow and insulin secretion while increasing oxidative stress in pancreatic cells, thereby promoting low inflammation, leading to insulin resistance and islet β cell impairment (150). iii. Regarding deteriorations in blood glucose levels among COVID-19 patients with previous T2DM, a recent study (151) reported that mRNA and protein expression levels of ACE2 in human islet β cells were significantly elevated under the actions of inflammatory cytokines, which may make β cells to be more vulnerable to severe infections.

##### Triggering Beta-Cell Autoimmunity

In addition to direct β-cell damage, SARS-CoV-2 invasion in pancreatic β-cells triggers β-cell autoimmunity in susceptible individuals, leading to development of type 1 diabetes (T1DM) (79).

##### SGLT1 Dysfunction

Sodium-glucose cotransporter 1 (SGLT1 or SLC5A1) is physiologically involved in the active absorption of glucose across the intestinal epithelium. ACE2-mediated SGLT1 imbalance in the intestinal epithelium may explain why COVID-19 aggravates diabetes complications and increases the risk of death (137, 152, 153). The existing data support the elevated expression levels of ACE2 in human gastrointestinal tissues (136), the high expressed ACE2 levels in intestinal mucosal cells and gallbladder makes these organs potential sites for viral entry and replication. Continuous viral replication at these ACE2 enrichment sites may be the basis for recurrence in some reported patients (believed to have been cured). ACE2-mediated downregulation of SGLT1 in the intestinal epithelium has been shown to prevent hyperglycemia in diabetic rats (154, 155). Although there is no direct evidence on effects of the combination of SARS-CoV-2 and ACE2 on its signaling cascade reactions, evidence from SARS-CoV-1 infections suggests that it can down-regulate ACE2 expressions (12), which may lead to up-regulation of SGLT1, thereby, aggravating hyperglycemia (154, 155). Since Ang II up-regulates while Ang-(1-7) suppresses the up-regulation of SGLT1 (154, 155), it is postulated that down-regulation of ACE2 expressions by binding SARS-CoV-2 leads to increased Ang II levels and to a corresponding decrease in Ang-(1-7) levels, which up-regulates SGLT1, increases intestinal glucose absorption, and promotes the development of hyperglycemia in COVID-19 patients.

##### Elevated Stress Levels

SARS-CoV-2 infections in diabetic patients may elevate stress levels and promote hyperglycemic hormone secretion, such as glucocorticoids and catecholamines, resulting in elevated glycemia and abnormal glucose variability (156).

##### Corticosteroid Treatment

Corticosteroids, including dexamethasone, are effective in preventing clinical deterioration and in reducing COVID-19-associated mortality rates. However, they are considered to be highly diabetogenic drugs, leading to uncontrolled hyperglycemia and even DKA or HHS.

#### Thrombotic Complications

Another feature of COVID-19 potential diabetogenic effect is diabetes-related endothelial dysfunction, which is characterized by hypercoagulable states, high incidences of thrombosis as well as microvascular complications.

SARS-CoV-2 infections activate immune responses, mediate the release of pro-inflammatory cytokines, over-activation of coagulation cascades as well as platelet aggregation, and induce micro- and macrovascular thrombosis, which are the main pathological characteristics of COVID-19. This greatly increases the likelihood of thromboembolic events, which are leading causes of death. Incidences of venous thromboembolism in COVID-19 patients are high (157). First, SARS-CoV-2 infections are associated with excess inflammation, and IL-6-related cytokine storms (158) can lead to high blood viscosity, thereby increasing the risk of stroke. Second, SARS-CoV-2 can also infect endothelial (54) and myocardial cells (136) through ACE2 receptors expressed in the vascular endothelium, myocardium and arterial smooth muscles, causing endothelial cell apoptosis, inflammatory cell infiltration, myocardial inflammation and injury, which increases the risk of thrombosis and stroke.

Hypercoagulable and fibrinolytic markers were found to be significantly increased in diabetic patients, and platelet activities as well as adhesion to endothelial walls were increased. These patients are at an increased risk of thromboembolic events or stroke (159, 160), especially in acute hyperglycemia (161) or high glucose variability states (162). Therefore, SARS-CoV-2 infections may promote their susceptibility to these conditions. Moreover, elevated levels of D-dimer, ferritin, IL-6 and other inflammatory markers in COVID-19 (163) may increase the risk of micro- and macrovascular complications in diabetic patients, which are as a result of low-grade vascular inflammation (164).

## Drug Therapy for Diabetes and Related-Complications in Patients With COVID-19

Diabetes and related metabolic complications are highly associated with severe COVID-19 development. Therefore, in addition to continuous strict blood glucose management to prevent acute complications **(** **Table 1** **)**, taking into account complications and specific thrombosis tendencies, diabetic patients with COVID-19 should be provided with appropriate antihypertensive, lipid-lowering and anticoagulant therapies to ensure better clinical outcomes. However, clinical administrations of glucocorticoids must weigh the beneficial effects of anti-inflammation against the potentially harmful effects of impaired immunity.

**Table 1:** A summary of classes of antidiabetic medications and their effects on ACE2 expression, inflammation, cardiovascular and kidney outcomes, effects in SARS-CoV-2 infection as well as issues prompting cautious use in patients with COVID-19.

Drugs	ACE2 expression	Anti-inflammatory	Cardiovascular and kidney outcome	Other beneficial effects	Effects in SARS-CoV-2 infection	Issue requiring caution in patients with COVID-19	Use in SARS-CoV-2 infection
Metformin	Increased expression and stability of ACE2	+	Benefit	i)Reduced uncomplicated DKA ii)Reduced microvascular complications and thrombotic events	i)Reduced disease severity and mortality ii)Improved prognosis iii)Potentially Inhibits virus-host protein interaction	i)Lactic acidosis ii)Deterioration of renal function iii)Gastrointestinal adverse events	Continue in hemodynamic stability and avoid in severe COVID-19 patients with hypoxia, hypoperfusion, septicemia, severe hepatic and renal impairment or hemodynamic instability
DPP-4 inhibitors	NR	+	Neutral	Reducing the risk of hypoglycemia	Not enough data (controversial, not defined)	i)Vildagliptin is associated with interstitial lung injury ii)Sitagliptin is associated with increased risk of venous thromboembolism	Continue in mild to moderate COVID-19 and avoid in critically ill
Thiazolidinediones	ADAM-17 down-regulation subsequently up-regulates mACE2	+	Benefit	i)Attenuates pulmonary fibrosis and ALI ii)Reducing the risk of thromboembolism events	Targeted 3CLpro and potentially inhibited SARS-CoV2 RNA synthesis and replication	i)Fluid retention ii)Peripheral edema iii)Heart failure	Discontinue in hospitalized COVID-19 patients with the risk of acute heart failure by current illness
GLP-1RAs	up-regulation mACE2	+	Benefit	i)Reduced hypoglycemia and glucose variability ii)Reducing the risk of AS and thromboembolism events	Not enough data	i)Gastrointestinal adverse events ii)Slow efficacy	May consider continuing in COVID-19 patients without associated gastrointestinal symptoms
SGLT2 inhibitors	up-regulation mACE2	+	Benefit	i)Reducing the risk of hypoglycemia ii)Reducing the risk of MACE and heart failure iii)Reduced all-cause mortality	i)Not enough data for disease prognosis ii)Inhibits cell Na+/H+ exchangers, reduces lactate serum levels, thus reducing cell acidosis and SARS-CoV-2 activation at acidic pH	i)DKA ii)Dehydration iii)kidney injury iv)genitourinary infection	Can be used in well-controlled, uncomplicated patients and avoid in moderate to severe patients requiring strict body fluid balance control
Insulin	down-regulation mACE2 correspondingly up-regulation sACE2	+	Neutral	Low risk of uncontrolled hyperglycemia and DKA	Not enough data (Possible increases the risk of poor prognosis and mortality)	i)Hypoglycaemia ii)weight gain	Can be used in patients requiring strict glycemic control
Sulfonylureas	NR	+	Inconclusive		Neutral	i)Hypoglycemia ii)Potential adverse cardiovascular effect	It is best to avoid using SUs, especially in critically ill patients
Glinides	NR	+	Neutral		Potentially inhibits the replication and transcription of SARS-CoV-2	The same as SUs	The same as SUs
Alpha-glucosidase inhibitors	NR	+	NR		Potentially inhibits virus-host protein interaction, inhibits the replication and transcription of SARS-CoV-2	Gastrointestinal adverse events	Avoid in patients with obvious gastrointestinal symptoms

NR, not report; ACE2, angiotensin-converting enzyme 2; DKA, diabetic ketoacidosis; COVID-19, coronavirus disease 2019; 3CLpro, 3-chymotrypsin-like protease; DPP4, dipeptidyl peptidase-4; SGLT2, sodium-glucose cotransporter 2; GLP-1RAs, glucagon-like peptide 1 receptor agonists; SARS‐CoV‐2, severe acute respiratory syndrome coronavirus‐2; MACE, major adverse cardiovascular events; AS, atherosclerosis.

### Antidiabetic Drugs

#### Metformin

The mechanisms of metformin involve AMPK-independent and AMPK-dependent pathways (165). Indirectly, metformin suppresses chronic inflammation by improving insulin resistance and hyperglycemia. One of the mechanisms through which it exerts its anti-inflammatory effects involves inhibition of AGEs formation, which will promote inflammation and glucose oxidation (166). Moreover, to exert its anti-proliferative and immunomodulatory effects, it inhibits nuclear factor-κB (NF-κB) through the AMP-activated protein kinase (AMPK) activation pathway (167–170). Besides, it regulates glucose and lipid metabolism (171). The activation of AMPK can also phosphorylate ACE2, which may alter the conformational and functional of the receptor, thereby reducing the binding of RBD to the ACE2 receptor (172), thus alleviating SARS-CoV-2 infections. Metformin up-regulates ACE2 expression and increases its stability in COVID-19.

A large-scale retrospective cohort study involving 6256 patients showed that compared to male patients, metformin treatment is significantly correlated with decreased mortality rates in female T2DM patients with COVID-19 (173). Other studies have supported the clinical benefits of metformin in diabetic patients with COVID-19 (174, 175).

Continuing metformin administration in hemodynamically stable COVID-19 patients may be safe, however, it should be avoided by critically ill patients with sepsis, severe liver and kidney damage, hypoxemia, inadequate perfusion or by those exhibiting hemodynamic instabilities due to the risk of lactic acidosis (176).

#### DPP4 Inhibitors

Apart from ACE2, DPP4 may also be involved in SARS-CoV-2 infections (62). It represents a potential target for mitigating the severity of COVID-19 by blocking viral transmissions and attenuating inflammatory responses. However, receptor inhibitors that are designed to lower glycemia may not apply to the binding interface between the virus and the receptor. Anti-DPP4 antibodies have been shown to inhibit hCoV-EMC infections of Huh-7 cells and human bronchial epithelial cells, while DPP-4is, such as vildagliptin, sitagliptin and saxagliptin were not shown to prevent this infection (60).

Rhee et al. reported that DPP-4i is associated with better clinical outcomes in COVID-19 patients (177). In a multicenter retrospective case-control study involving 338 consecutive T2DM patients with COVID-19, sitagliptin therapy reduced mortality and improved clinical outcomes (178). Mirani et al. (179) reported that DPP-4is are significantly and independently correlated with a low mortality risk. Diabetic patients administered with DPP-4i exhibited a lower severity of COVID-19, a lower risk of mortality as well as a lower need for mechanical ventilation. Multiple retrospective case-control studies have shown that DPP-4i does not affect the risks of hospitalization, ICU admission, mortality and clinical outcomes in T2DM patients with COVID-19 (175, 180). Currently, evidence for the use of DPP-4i in diabetic patients with COVID-19 is inconclusive. Two ongoing clinical trials are assessing whether addition of DPP-4 inhibitors on the basis of insulin therapy can help improve the severity of COVID-19 (NCT04341935; NCT04542213), which may also be extended to patients without diabetes mellitus.

DPP-4i mainly affects postprandial blood glucose levels and lowers hypoglycemia risk. In stable patients with good food intake, DPP-4i can be continued (139), however, it should be suspended in patients with severe discomforts (181). DPP-4i can be administered in impaired renal function patients, however, for COVID-19 patients who clinically manifest fluid depletion or systemic sepsis, DPP-4i doses should be adjusted for optimal outcomes. Sitagliptin is associated with an increased risk of venous thromboembolism, which may require discontinuation in hospitalized COVID-19 patients (182).

#### Thiazolidinedione

Pioglitazone downregulates ADAM-17, a mACE2 cleaving enzyme in the human skeletal muscles, leading to elevated mACE2 levels. Moreover, pioglitazone has been shown to elevate ACE2 expression in animal models, especially in the liver and adipose tissues (183, 184), raising concerns about increased susceptibility to SARS-CoV-2 infection. Computer-based virtual bioinformatics analysis revealed that pioglitazone might target 3-chymotrypsin-like protease (3CLpro) to inhibit SARS-CoV-2 RNA synthesis and replication (185).

Thiazolidinediones (such as pioglitazone and rosiglitazone) have the potential risk of fluid retention and peripheral edema, which may increase the risk of heart failure. Therefore, they should be avoided in COVID-19 patients with a risk of acute heart failure from existing diseases (186). The antihyperglycemic effects of thiazolidinedione last for several weeks after drug withdrawal (just like the effect of maintaining fluid), therefore, temporary interruption of treatment has little effect on glycemic control (187). More clinical trials should be performed to optimize the risk-benefit ratio of pioglitazone in COVID-19 patients.

#### GLP-1 Receptor Agonist

GLP-1RAs have been reported to increase ACE2 expressions in the lungs and hearts of type 1 diabetic rats (188), therefore, it can offset the down-regulation effects of diabetes on the expressions of ACE2 in the lungs. However, it has not been established whether it can affect clinical progressions of COVID-19. Indeed, clinical evidence for GLP-1RAs administration in SARS-CoV-2 infections is inconclusive. Given the beneficial effects of GLP-1RA (189) in prevention of cardiovascular and kidney diseases have been defined (190), these drugs may be a priority for treatment of diabetic patients with this risk. GLP-1RAs treatment can lower hypoglycemia risk, reduce glucose variability as well as catabolism by suppressing glucagon levels (191), which may exert a protective effect on critically ill patients in the ICU. However, it is not recommended to start or maintain such therapies in acute or critical situations (e.g. severe COVID-19), as they may work slowly and are associated with increased gastrointestinal adverse events. Studies in animal models have reported that GLP1 analogues can inhibit atherosclerosis formation, stabilize the plaque of carotid artery and aortic arch (189, 192). The REWIND study also showed that dulaglutide can decrease stroke incidences in T2DM patients (193). Taken together, it will be beneficial for diabetic patients with COVID-19 to be administered with antidiabetic drugs that can reduce the risk of thromboembolism.

#### SGLT2 Inhibitors

Evidence suggests that SGLT-2i promotes the expression of ACE2 in the heart and kidneys (194), and then increases Ang1-7 levels. Ang1-7 can effectively expand blood vessels, exert antioxidant and anti-fibrotic effects, and is involved in attenuation of cytokine storms as well as in prevention of ARDS, which is also considered to be a possible heart and kidney protection mechanism for these drugs. Although SGLT-2i alleviates inflammatory injury and endothelial dysfunction, its potential beneficial effects in diabetic patients with COVID-19 have not been fully established. Evidence from DARE-19 (195), a randomized, double-blind, placebo-controlled study of dapagliflozin for delaying disease progression and prevention of major clinical events in hospitalized COVID-19 patients with cardiometabolic risk factors, clarified that dapagliflozin treatment did not significantly improve clinical recovery or reduce the risk of organ dysfunction as well as death, but was well tolerated.

SGLT-2i has important cardiovascular and renal protective functions and may protect important organs during COVID-19. Similarly, the results from the DARE-19 study do not support the conventional discontinuation of dapagliflozin in COVID-19. Concerns about the administration of SGLT2is in COVID-19 are associated with reduced blood volume, increased risk of renal insufficiency, genitourinary tract infections and ketoacidosis. DKA with normal blood glucose has been reported in SGLT-2i administered T1DM and T2DM patients (196, 197). Drug withdrawal must be considered if glomerular filtration rate decreases and renal function deteriorates. SGLT-2i is associated with dehydration and anorexia, therefore, it is not recommended for diabetic patients with moderate to severe COVID-19 who need strict body fluid balance control in conditions of inadequate food intake, dehydration and insufficient blood volume.

#### Insulin

Insulin is widely used in COVID-19 patients with hyperglycemia, especially in severe cases. However, recent conflicting evidence suggests that insulin therapy may contribute to the death of diabetic patients with COVID-19. A retrospective study involving 689 T2DM patients with COVID-19 reported that insulin therapy is significantly associated with increased mortality, accompanied by aggravated systemic inflammation and exacerbated damage to vital organs (198). Nonetheless, residual confounders cannot be ruled out. Patients receiving insulin therapy are usually in severe conditions, and have high blood glucose levels, therefore insulin is preferred.

Immunofluorescence analysis confirmed elevated ACE2 and ADAM17 levels in the kidneys of diabetic Akita mice and decreased ACE2 levels in the kidneys of insulin-treated Akita mice (199). Helena et al. (84) reported that insulin down-regulated the expression of mACE2 in non-obese diabetic mice. In a longer follow-up period, insulin administration restored serum sACE2 levels. At near-physiological concentrations, sACE2 enhances SARS-CoV-2 infection, and it may be a potential biomarker for COVID-19 severity. Therefore, increased severity of COVID-19 after insulin administration may be partly attributed to the ACE2 receptor. However, evidence for these outcomes is limited. Large, prospective, randomized, placebo-controlled clinical trials should be performed to elucidate on the harmful effects of insulin therapy in COVID-19 patients. Tight glycemic control is important because poor-outcomes are correlated with high blood glucose levels in COVID-19. Taken in sum, fuzzy evidence on insulin should not prevent its use in hospitalized patients that are in need of tight glycemic control.

#### Sulfonylureas (SUs)

Feng et al. (200) predicted that glibenclamide (SUs) and tolazamide (the first generation of sulfonylurea drugs) might be used to treat COVID-19 coexisted with T2DM, but their findings, which are based on computer simulations, need *in vitro* experimental validation. Multiple studies have reported that SUs are harmless or unhelpful to COVID-19 patients (105, 201–203), while some studies report that SUs administration may lead to poor prognostic outcomes in patients with myocardial infarction (MI) (204). On the contrary, some studies have refuted the association between SUs and MI-induced death (205, 206). Besides, SUs increase the risk of hypoglycemia, which is worsened in patients with impaired renal functions or insufficient calorie intakes. Given the risk of severe hypoglycemia and cardiovascular safety, SUs should be avoided by COVID-19 patients, especially critically ill patients.

#### Glinides and Alpha-Glucosidase Inhibitors

Through virtual screening, repaglinide (207) and acarbose (208, 209) were found to inhibit SARS-CoV-2 replication and transcription. However, molecular structure information differs from clinical effectiveness, therefore, careful interpretation is needed. The action mode of glinides is similar to that of SUs, therefore, they should be cautiously administered, especially in COVID-19 patients with related cardiac injuries. Given the gastrointestinal side effects, α-glucosidase inhibitors are not suitable for COVID-19 patients with obvious gastrointestinal symptoms.

### Therapeutic Options for Related-Complications

#### ACE Inhibitor (ACEi) and Angiotensin Receptor Blockers (ARBs)

Previous report had shown that lisinopril and losartan are associated with a significant increase in ACE2 levels (210). Concerns raised about the up-regulation of ACE2 expression might increase the risk of COVID-19 infection. Contrarily, it has been proposed that elevated ACE2 levels after RAAS inhibitor treatments might actually be beneficial for COVID-19 induced lung injury (211). Elevated mACE2 levels are associated with upregulated Ang1-7, which has vasodilation, anti-fibrosis as well as protective effects on lung injury (212). However, SARS-CoV-2 infection down-regulates mACE2, leading to over-accumulation of AngII toxicity, which may aggravate inflammation and thrombosis (13), and lead to more severe lung injury (213).

Through feedback regulation, the accumulation of Ang II in COVID-19 patients may lead to low mRNA expression levels of ACE2. Gallagher et al. reported that Ang II over-expression reduces ACE2 mRNA expressions in rat cardiomyocytes and fibroblasts, which was blocked by losartan, an ARB (214).

With aging and disease, the ACE-Ang-II-AT1R axis controls ACE2 - Ang (1-7) -MasR axis. After SARS-CoV-2 infection, virus-mediated endocytosis further reduces mACE2 expressions, resulting in Ang II surge, thereby triggering another round of mACE2 reduction through up-regulation of ADAM-17-mediated shedding by AT1R. Therefore, in COVID-19 patients with comorbidities, viral entry and Ang II accumulation downregulates ACE2, which transfers RAS balance to the harmful end. ARBs and ACEI turn the balance to the beneficial axis, attenuate Ang II accumulation, and prevent AT1R-mediated mACE2 loss as well as harmful cascades. Due to the administration of ARB and ACEI, this recovered ACE2 was mistakenly considered to be over-expressed.

Losartan was shown to prevent severe lung injury and pulmonary edema in ACE2 knockout mice (215). A retrospective study of hospitalized patients with COVID-19 and hypertension showed that ACEI and ARBs ameliorated clinical outcomes and alleviated cytokine storms (216, 217). A multicenter retrospective study involving 1128 adult hypertensive patients diagnosed with COVID-19 showed that hospitalized patients with ACEI/ARB had a lower risk of all-cause death compared to those without ACEI/ARB (218). A recent meta-analysis suggested that ACEI therapy reduced the risk of SARS-CoV-2 infection and that blocking RAS might reduce all-cause mortality in COVID-19 patients (219). Multicenter, blinded RCTs on the impacts of losartan on clinical prognosis of COVID-19 patients who need/do not need hospitalization (NCT04312009, NCT04311177), and the ongoing trials on whether the lead-in of ACEi in* de novo* may help ameliorate COVID-19 outcomes (NCT04366050) will elucidate on these aspects.

Experts strongly recommend treatment continuation with ACEi and ARBs. Substantial evidence indicates that RAAS inhibitors elevate the expressions of ACE2, but they are not associated with increased SARS-CoV-2 infection risks or poor prognosis of COVID-19 (220–223), on the contrary, it is even helpful to some extent. A sudden withdrawal of ACEI/ARB may cause greater harm, especially in patients with a high risk of renal and cardiovascular diseases. Accordingly, to maintain their antihypertensive and cardiorenal protective effects in COVID-19 patients, it is not recommended to discontinue these drugs.

#### Aspirin

Due to reports on acute respiratory tract infections previously, concerns were raised about the possibility of increased risks of adverse events (such as multiple organ failure, ARDS and death) induced by NSAID treatments in COVID-19 patients (224, 225). Nonetheless, given the lack of evidence that they may lead to serious adverse consequences in COVID-19, major scientific societies worldwide have issued advisories to discourage either avoidance or suspension of NSAIDs in COVID-19 (226–228).

On the contrary, aspirin, a non-steroidal anti-inflammatory drug, has various pharmacological properties that can exert potential beneficial effects for COVID-19 patients. First, its analgesic and antipyretic effects may help alleviate the specific symptoms of COVID-19. Second, it may exert anti-inflammatory, antithrombotic and antiviral effects (229–233), which may be useful in preventing pathophysiological processes of severe clinical manifestations involved in COVID-19. Much solid evidence from *in vitro* and experimental models support that aspirin reduces the synthesis and replication of several RNA-encapsulated viruses (including human CoV-229E and MERS-coronavirus) in infected cells, thereby reducing viral titers and virulence (229). Moreover, excess Ang II signaling in COVID-19 may activate the STING pathway (234), which promotes hypercoagulation through the secretion of interferon-β and tissue factors by monocyte-macrophages. Aspirin has been found to directly inhibit the STING pathway (235), thereby reducing tissue factor procoagulant activities.

Aspirin improves survival outcomes for patients with different types of infections, which is characterized by hyperactivation of inflammatory cascades and increased platelet reactivities (236–238). Zhou et al. reported that COVID-19 severity can be alleviated by aspirin. After investigating a cohort of adult COVID-19 patients admitted to several hospitals in the United States, aspirin administration was found to reduce mechanical ventilation rate, ICU occupancy rate and hospital mortality rates (239). A cross-sectional study using data from the Leumit Health Services database observed that the possibility of COVID-19 infection, disease duration and mortality are negatively correlated with aspirin administration for primary prevention (240).

Prophylactic anticoagulation is a potential conventional treatment option for diabetic patients with COVID-19. For hospitalized patients with moderate to severe COVID-19, initiation of anticoagulant therapy may be prudent, but may not be necessary for mild patients. Anticoagulant therapy (e.g., aspirin, low molecular weight heparin) for severe COVID-19 patients with a high risk of thromboembolism (e.g., D-dimer increased patients) (241) has been associated with better prognostic outcomes. A randomized clinical trial, RECOVERY II, is underway to test the effectiveness of low-dose aspirin as an antithrombotic and anti-inflammatory option for COVID-19 patients (242). Given the increased risk of thromboembolism among patients with COVID-19 and diabetes, we recommend that physicians consider the administration of antiplatelet or anticoagulant therapies more actively.

#### Heparin

Heparin sulfate (HS) promotes the recruitment of SARS-CoV-2 to the cell surface, thereby increasing its local concentrations to effectively bind ACE2 and increase viral entry (243, 244). The SARS-CoV-2 spike protein can simultaneously bind heparin and ACE2. Therefore, heparin has the ability to compete with HS for SARS-CoV-2 S protein binding, which is important in inhibiting SARS-CoV-2 infection.

COVID-19 patients usually present with thrombosis complications, including microthrombosis, venous thromboembolism and stroke, and often receive therapeutic unfractionated heparin (UFH) or low molecular weight heparin. Both drugs can block viral infection (244, 245). Plasma heparin levels of 0.3 – 0.7 unit/mL (equivalent to 1.6-4 μg/mL) can achieve effective anticoagulation, and this concentration is sufficient to prevent the S protein from binding cells. However, it does not prevent SARS-CoV-2 infection, but rather, attenuates infection according to viral load (243). Plasma heparin levels of ≥ 0.5 μg/mL can effectively reduce SARS-CoV-2 infection by more than 4 times. UFH and degradation of cell surface HS was shown to reduce infection by more than 5 times without affecting cell viability (243). These findings further emphasize on the potential of using UFH or other non-anticoagulant heparins to prevent SARS-CoV-2 adhesion.

#### Statins

Statins have been shown to restore the lowered ACE2 levels that are suppressed by high lipids such as low-density lipoprotein or lipoprotein(a) (246). Upregulated ACE2 levels may help alleviate multiple organ injuries after SARS-CoV-2 infections.

Besides, lipid-lowering effects of statins can improve hyperlipidemia associated with antiretroviral and immunosuppressive drugs that are based on protease inhibitors in COVID-19.

The lipid-lowering effect of statins is beneficial for cardiovascular diseases. Besides, statins modulate immune responses and alleviate inflammation as well as oxidative stress, which may help in attenuating cytokine storms. MYD88, a protein connector of the downstream inflammatory signaling pathways of Toll-like receptor and IL-1 receptor family members, plays a key role in activation and amplification of innate immune responses (247). SARS-CoV-1 induces inflammatory host responses by activating the Toll-like receptor (TLR)-MYD88-NF-κB pathway (248). Studies involving animal models have shown that statins inhibit myeloid differentiation of MYD88 and NF-κB activation. Inhibition of MYD88 was shown to suppress pneumonia-associated damage and improved the survival rate of mice infected with SARS-CoV and MERS-CoV (249).

Elevated C-reactive protein (CRP) levels are a risk factor for increased mortality in diabetic patients with COVID-19 (175). In individuals without COVID-19 or hyperlipidemia but with elevated CRP levels, rosuvastatin was found to decrease the risk of major cardiovascular events by 44% (250), which was attributed to its pleiotropic anti-inflammatory effects (251). A Chinese study reported that statins administration was correlated with low risk of all-cause mortality and good recovery characteristics in hospitalized patients with COVID-19 (252). Nevertheless, clinical benefits of statins should be further verified in RCTs.

The regulation of ACE2 expression affects SARS-CoV-2 infection and disease-associated mortality. Since the long-term benefits of statins may include weakening cytokine storms by suppressing IL-6 and IL-1β levels, it should not be discontinued. Moreover, COVID-19 is associated with higher cardiovascular-related mortality rates, particularly in patients with higher risk factors (including hypertension and T2DM). In these patients, statins can be administered to maintain or optimize lipid management and improve endothelial dysfunctions (253). Given the risk of hyperlipidemia, which is associated with antiretroviral immunosuppressive drugs, and cardiovascular disease, we recommend diabetic COVID-19 patients continuing the current statin therapy. Most statins are metabolized by CYP3A4 in the liver, however, in cases of simultaneous administrations of CYP3A4 inhibitors for COVID-19, such as ritonavir, it is recommended to start with lower doses of statins, while monitoring creatine kinase and transaminase levels.

#### Corticosteroids

Corticosteroids play an important role in the treatment of ARDS and sepsis, which are also the therapeutic targets for severe COVID-19 infections. COVID-19 is characterized by strong inflammatory responses. Corticosteroids may play an important role in inhibiting inflammation in the lungs and other tissues, thereby regulating inflammation-mediated lung injury and reducing the risk of ARDS as well as death by attenuating cytokine production.

A recent meta-analysis revealed that systemic glucocorticoid therapy reduces short-term all-cause mortality in severe COVID-19 patients (254). The RECOVERY study reported that dexamethasone administration reduced mortality by 36% (95% CI 0.5 - 0.81) in hospitalized COVID-19 patients receiving invasive mechanical ventilation (255). However, there are conflicting findings. Glucocorticoids therapy in COVID-19 patients was shown to induce delayed viral RNA clearance, increased mortality and incidences of complications (256). Moreover, glucocorticoids administration in diabetes patients with COVID-19 has been associated with poor prognostic outcomes, and meanwhile, the results also confirmed the harmful effect of hyperglycemia on disease outcomes (257, 258). The possible explanation is that, systemic corticosteroids therapy may decrease the expression of Ang1-7 and Mas receptors, leading to deterioration of hyperglycemia, which aggravates metabolic control (259). The possibility that hyperglycemia offsets mortality benefits of systemic corticosteroids cannot, therefore, be ruled out. Effectiveness of corticosteroid therapies in diabetic patients with COVID-19 has not been established, however, further long-term studies are needed to confirm this result.

Although corticosteroids reduce lung inflammation, they also inhibit immunity and pathogen clearance (256). Viral shedding after SARS-CoV-2 infection seems to be high in the early stages, and then decreases (260–262). Glucocorticoid treatment might be ineffective or even harmful in the early stages of infection (by increasing viral load), therefore, it is not recommended to use corticosteroids at the outset of the COVID-19 pandemic. For 1-week post-SARS-CoV-2 infection patients, or those receiving respiratory support, dexamethasone has been shown to have greater survival benefits, indicating that inflammation is predominant and viral replication is secondary at this stage. The WHO provisional guidance on clinical management of severe acute respiratory infections induced by SARS-CoV-2 suggests that corticosteroids should not be used outside clinical trials.

## Conclusions and Perspectives

This review preliminarily summarizes current research progress on receptors related to COVID-19 with diabetes, especially ACE2. The mutual physiological effects between COVID-19 and diabetes, and commonly used medications have been updated and summarized. More RCT studies in the future will enable us to have a more accurate understanding of these findings. Optimal and timely blood glucose management can improve the clinical course of diabetic patients with COVID-19. Insulin is the preferred hypoglycemic agent for hospitalized and critically ill patients, however, the effects of insulin on ACE2 expressions and COVID-19 progression remains uncertain. For patients with COVID-19, AT1 receptor blocker treatment should not be interrupted. Considering that ACE2 autoantibodies increase the severity of COVID-19, it raises an important question whether AT1 receptor blockers or ACEI should be initiated for patients at risk of infection or diagnosed with COVID-19.

The biological functions of sACE2 (protective or risk factor biomarkers) in COVID-19 are poorly understood. The reasons as to why mACE2 proteins vary between different genders and ages leaving much to be explored. Whether for effective antiviral therapies, vaccines development or the progression of diabetes as well as related complications, ACE2 will remain the target for research as well as drug development for COVID-19 in the future. ACE2 is a complex site for diabetic nephropathy and can affect its progression, however, it has not been determined whether SARS-CoV-2 infection accelerates diabetic nephropathy progression through ACE2. In particular, long-term proteinuria and GFR monitoring should be performed for COVID-19 patients with diabetes. With genetic variations in the novel coronavirus, in order to contain its spread and reduce its associated severity and mortality, efforts should be aimed at developing effective vaccines. In this regard, it may be necessary to determine whether therapeutic options for diabetic patients have any impact on antibody responses and antibody levels of various vaccines for COVID-19. Besides, the impact of these vaccines on diabetic patients remains to be further explored.

## Author Contributions

LX drafted and wrote the manuscript. ZZ revised the manuscript. QW, YC, and DL reviewed the manuscript and provided critical comments. WW conceived and organized the work, and revised the manuscript. All authors contributed to the article and approved the submitted version.

## Conflict of Interest

The authors declare that the research was conducted in the absence of any commercial or financial relationships that could be construed as a potential conflict of interest.

## Publisher’s Note

All claims expressed in this article are solely those of the authors and do not necessarily represent those of their affiliated organizations, or those of the publisher, the editors and the reviewers. Any product that may be evaluated in this article, or claim that may be made by its manufacturer, is not guaranteed or endorsed by the publisher.
